# Chitin Deacetylase Homologous Gene *cda* Contributes to Development and Aflatoxin Synthesis in *Aspergillus flavus*

**DOI:** 10.3390/toxins16050217

**Published:** 2024-05-09

**Authors:** Xin Zhang, Meifang Wen, Guoqi Li, Shihua Wang

**Affiliations:** State Key Laboratory of Ecological Pest Control for Fujian and Taiwan Crops, Key Laboratory of Pathogenic, Fungi and Mycotoxins of Fujian Province, School of Life Sciences, Fujian Agriculture and Forestry University, Fuzhou 350002, China; zhangxin5319@gmail.com (X.Z.); meifangwen@hotmail.com (M.W.); liguoqi@fafu.edu.cn (G.L.)

**Keywords:** *Aspergillus flavus*, aflatoxin, chitin deacetylase, *cda6*

## Abstract

The fungal cell wall serves as the primary interface between fungi and their external environment, providing protection and facilitating interactions with the surroundings. Chitin is a vital structural element in fungal cell wall. Chitin deacetylase (CDA) can transform chitin into chitosan through deacetylation, providing various biological functions across fungal species. Although this modification is widespread in fungi, the biological functions of CDA enzymes in *Aspergillus flavus* remain largely unexplored. In this study, we aimed to investigate the biofunctions of the CDA family in *A. flavus*. The *A. flavus* genome contains six annotated putative chitin deacetylases. We constructed knockout strains targeting each member of the CDA family, including Δ*cda1*, Δ*cda2*, Δ*cda3*, Δ*cda4*, Δ*cda5*, and Δ*cda6*. Functional analyses revealed that the deletion of CDA family members neither significantly affects the chitin content nor exhibits the expected chitin deacetylation function in *A. flavus*. However, the Δ*cda6* strain displayed distinct phenotypic characteristics compared to the wild-type (WT), including an increased conidia count, decreased mycelium production, heightened aflatoxin production, and impaired seed colonization. Subcellular localization experiments indicated the cellular localization of CDA6 protein within the cell wall of *A. flavus* filaments. Moreover, our findings highlight the significance of the CBD1 and CBD2 structural domains in mediating the functional role of the CDA6 protein. Overall, we analyzed the gene functions of CDA family in *A. flavus*, which contribute to a deeper understanding of the mechanisms underlying aflatoxin contamination and lay the groundwork for potential biocontrol strategies targeting *A. flavus*.

## 1. Introduction

*Aspergillus flavus* is a fungus of the genus *Aspergillus* that is widely distributed and highly adaptable to its environment [1]. However, under improper storage conditions, *A. flavus* can proliferate in various crop seeds and produce aflatoxins [2]. On one hand, *A. flavus* can cause poison in humans and animals, leading to tumors, cancer, and even death [3,4]. On the other hand, *A. flavus* spores or mycelium can infect host tissues and cause invasive pulmonary aspergillosis and infectious aspergillosis in animals [5]. Moreover, *A. flavus* is the primary contaminant and major producer of AFB_1_, which is one of the most hazardous carcinogenic toxins for humans and animals [6]. In summary, *A. flavus* poses a significant threat to food security, human, and animal health.

The fungal cell wall, comprising β-1, 6-glucan, β-1, 3-glucan, and chitin, represents a pivotal structure at the interface between fungi and their external surroundings [7]. Its composition dynamically changes during various growth and differentiation stages, exerting crucial roles in fungal survival, growth, and interaction with the environment [8]. Notably, the fungal cell wall safeguards cellular integrity, facilitates intercellular adhesion, and mediates cell–substrate attachment. Moreover, it significantly influences the pathogenicity and virulence of invasive fungi [9]. Due to the importance of the cell wall to fungi, it serves as a therapeutic target in clinical research and development of antifungal drugs [10,11].

Chitin is a crucial component of the cell wall for fungi, serving to maintain its stability. Chitin deacetylase (CDA) is prevalent across fungal species, and catalyzes the conversion of chitin to chitosan, modulating cell wall formation, integrity, and pathogenicity [12,13]. Structural biology studies have shown that CDAs share a similar catalytic mechanism with other members of the carbohydrate esterase 4 (CE4) family. Previous investigations conducted in model fungi like *Saccharomyces cerevisiae* and *Cryptococcus neoformans* have illuminated the essentiality of CDAs. In *S. cerevisiae*, the structure of chitosan is essential for spores to maintain their structural stiffness and resist various environmental pressures [13]. Two genes, *cda1* and *cda2*, play vital roles in the formation of correct ascospore walls in *S. cerevisiae* [14]. In *C. neoformans*, which harbors four *cda* genes, namely *cda1*, *cda2*, *cda3*, and *fpd1*, deletion of *cda* genes affects the chitosan content of the cell wall [15]. Deacetylated chitosan serves as a crucial structural component of the trophic mycelium cell wall of *C. neoformans*, contributing to the maintenance of structural integrity, with the enzyme activity of Cda1 being critical to the pathogenesis of this species [16]. In *Pyricularia oryzae,* the chitin deacetylase PoCda7 is essential for the pathogenicity of the rice blast fungus [17]. There are seven *cda* genes in the *A. fumigatus* genome, of which Cod4 and Cod7 play significant roles in polarity abnormality and conidiation [18]. While the seven candidate CDA proteins in *A. fumigatus* exhibit minor contributions to fungal cell wall synthesis and virulence, Cda2 is an exception, involved in conidiation [19]. The above results showed that CDAs play distinct roles in conidiation, cell wall formation, and pathogenicity across various fungal species.

All members of the CDA family proteins harbor conserved domains, including the NodB homology domain, pivotal for chitin deacetylation. The NodB domain is the landmark domain of carbohydrate esterase 4 (CE4) family, which can convert chitin into chitosan in various fungi. In *Rhizobium,* the NodB protein is a chitosan deacetylase capable of deacetylating the non-reducing N-acetylglucosamine residues of chitosan, but not the monosaccharide N-acetylglucosamine [20]. Furthermore, additional domains like chitin-binding domains (CBDs) and glycosylphosphatidylinositol (GPI) anchoring motifs enhance CDA functionality by facilitating cell surface interactions and membrane anchorage [21]. The GPI is involved in attaching protein to the fungal cell wall and cell membrane [22,23]. Notably, the CDA homologous gene of the *CBP1* gene in *Magnaporthe grisea* contains a chitin-binding domain (CBD) and the NodB domain, which is critical in response to external hydrophobic signals [24].

Based on studies in phytopathogenic filamentous fungi, we hypothesized that CDAs might play important roles in conidiation, aflatoxin biosynthesis, and host–pathogen interactions in *A. flavus*. In this study, we aim to comprehensively investigate the impact of the chitin deacetylase family on various physiological and pathological aspects of *A. flavus*. Another critical aspect of this research is to assess the impact of CDAs on the synthesis of aflatoxins in *A. flavus*. This study also investigated how the CDA family influences pathogenicity in *A. flavus*. In conclusion, this study aims to establish a theoretical framework for the prevention and management of *A. flavus*.

## 2. Results

### 2.1. Bioinformatics Analysis of CDA Family in A. flavus

**Figure 1:**
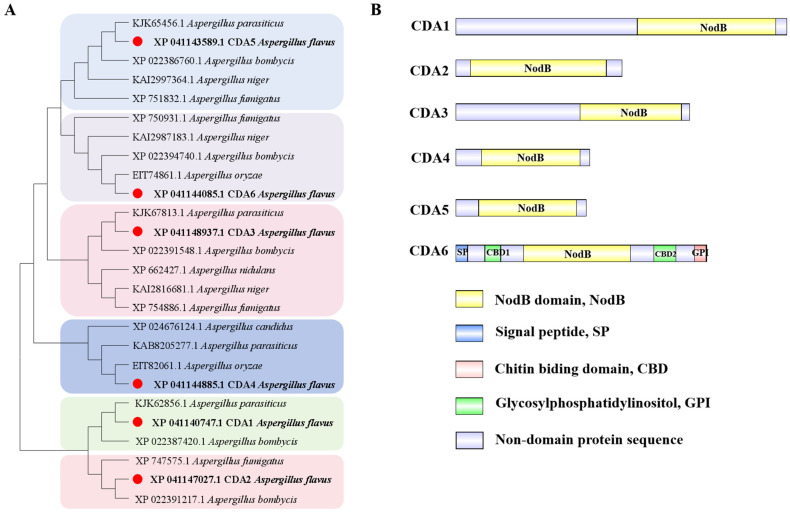
Bioinformatics analysis of CDA family in *A. flavus*. (**A**) Phylogenetic tree construction of CDA homologous proteins between different species. The red bullet represents *A. flavus* CDAs. (**B**) Domain analysis of CDA family in *A. flavus*.

Through homology comparison, we identified and named six chitin deacetylase genes in *A. flavus* as *cda1*, *cda2*, *cda3*, *cda4*, *cda5*, and *cda6*, respectively (Table S1). Protein sequences of CDA family in *A. flavus* were compared with the homologous sequences from *A. nidulans*, *A. fumigatus*, *A. niger*, *A. parasiticus*, and *A. oryzae*. Phylogenetic analysis revealed the presence of homologous proteins of CDA members across various *Aspergillus* genera. Notably, the highest percent identity (97.42%) was observed between *A. flavus* CDA6 (XP 041144085.1) and *A. oryzae* (EIT74861.1), underscoring the high conservation of the CDA family within *Aspergillus* species (Figure 1A). All members of the CDA family contain a conserved NodB homology domain, characteristic of the carbohydrate esterase 4 (CE4) family. This domain catalyzes the conversion of chitin to chitosan in diverse fungi. Additionally, the CDA6 protein contains chitin-binding domains (CBDs), a signal peptide (SP), and a glycosylphosphatidylinositol (GPI)-anchoring domain (Figure 1B).

### 2.2. Quantitative Analysis of Chitin in A. flavus

**Figure 2:**
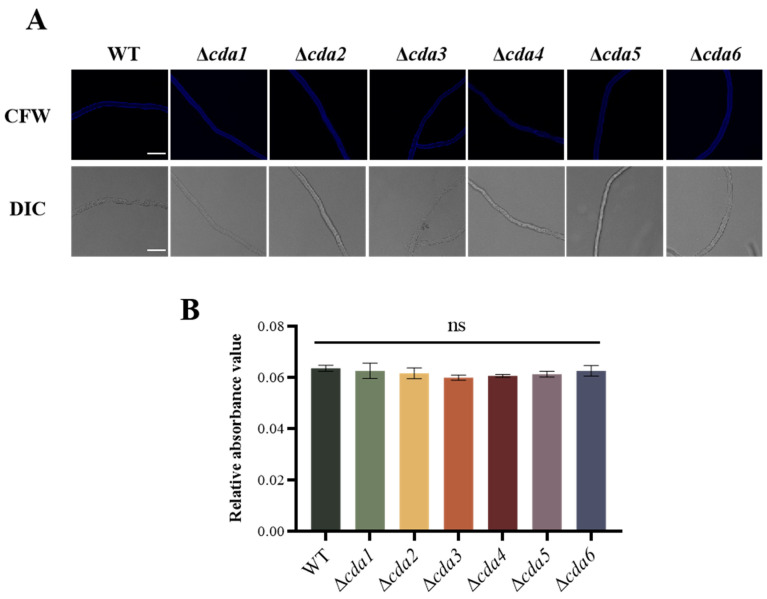
Quantitative analysis of chitin in *cda* single deletion strain of *A. flavus*. (**A**) Confocal microscope results of hyphae from Δ*cda1*, Δ*cda2*, Δ*cda3*, Δ*cda4*, Δ*cda5*, and Δ*cda6* dyed by CFW (CFW, Fluorescent Brightener 28, can bind to chitin and fluoresces blue. Bar = 25 μm). (**B**) Quantitative analysis of chitin in *cda* single knockout strain of *A. flavus* by chemical extraction. ns indicates no significance.

To investigate the biological functions of CDA members in *A. flavus*, gene deletion strains for *cda1*, *cda2*, *cda3*, *cda4*, *cda5*, and *cda6* were constructed using homologous recombination. After PCR verification, these strains were subjected to further testing (Figure S1). Given the high homology of CDA family members with those of other fungi, it was hypothesized that *A. flavus* CDA proteins may have chitin deacetylation activity. Quantitative analysis of chitin using Calcofluor White (CFW) staining revealed blue fluorescence on all mycelial cell walls. However, the fluorescence intensity of single *cda* knockout strains did not significantly differ from that of the WT strain (Figure 2A). The absorbance values of the developed color with p-dimethylaminobenzaldehyde were measured, and the data were analyzed for the relative content of cell wall chitin in *A. flavus*. The results showed no significant difference in chitin content between Δ*cda1*, Δ*cda2*, Δ*cda3*, Δ*cda4*, Δ*cda5*, and Δ*cda6* strains compared to WT strain (Figure 2B), indicating that deletion of *cda* genes did not affect chitin content. In summary, single *cda* gene deletion within the CDA family did not influence the chitin content of *A. flavus* cell wall.

### 2.3. Involvement of CDA Members in Regulating Conidial Formation in A. flavus

**Figure 3:**
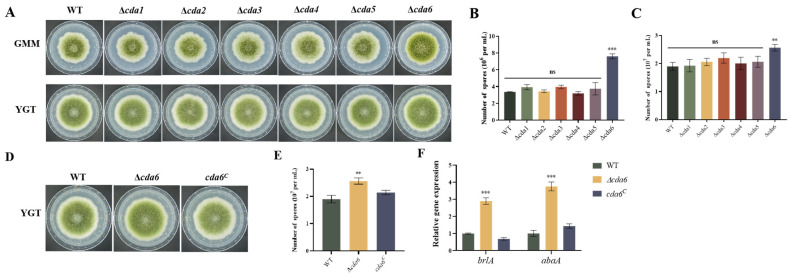
Role of CDA family members in radial growth and sporulation of *A. flavus*. (**A**) WT, Δ*cda1*, Δ*cda2*, Δ*cda3*, Δ*cda4*, Δ*cda5*, and Δ*cda6* strains were inoculated in GMM and YGT medium respectively to observe the growth status. (**B**) Statistics of conidial number of the above strains in GMM medium. (**C**) Statistics of conidial number of the above strains in YGT medium. (**D**) Growth status of WT, Δ*cda6*, and *cda6^C^* strains on YGT medium. (**E**) Statistics of conidial number of WT, Δ*cda6*, and *cda6^C^* in YGT medium. (**F**) Relative expression levels of the conidial formation regulatory genes *brlA* and *abaA* in the above strains. ns indicates not significant, ** indicates that the significance level was *p* < 0.01, and *** indicates that the significance level was *p* < 0.001 (*n* = 3).

To test the role of CDA family members in conidial formation in *A. flavus*, spore suspensions of WT, Δ*cda1*, Δ*cda2*, Δ*cda3*, Δ*cda4*, Δ*cda5*, and Δ*cda6* strains were inoculated on GMM basal medium and YGT nutrient-rich medium. The colony diameters of CDA member knockout strains in both GMM and YGT media were not significantly different compared to WT strain (Figure 3A), indicating that CDA family members do not regulate radial growth of *A. flavus*. However, the results revealed a significant increase in spore production in the Δ*cda6* strain compared to WT (Figure 3B,C), indicating involvement of the *cda6* gene in regulating conidial formation.

To further investigate the biofunctions of *cda6* gene in *A. flavus*, we also constructed a complement strain of this gene (*cda6^C^*). Subsequently, WT, Δ*cda6*, and *cda6^C^* strains were further incubated in YGT media at 37 °C for 5 days. The result in Figure 3D,E indicated that the *cda6* gene negatively regulates the spore formation in *A. flavus,* and the complemented strain *cda6^C^* fully restored its function. To investigate the signaling pathway of the *cda6* gene regulating conidial formation in *A. flavus*, the transcript levels of regulatory genes for spore formation, *brlA* and *abaA*, were examined by qPCR. The results showed that the relative expression of *brlA* and *abaA* genes was significantly up-regulated in the Δ*cda6* strain compared to the WT strain (Figure 3F), indicating that the *cda6* gene regulated spore formation by affecting the transcript levels of the *brlA* and *abaA* genes.

### 2.4. Fluorescence Localization of CDA6 Protein in A. flavus

**Figure 4:**
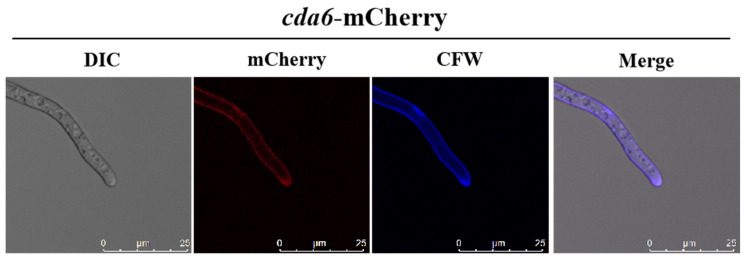
Subcellular localization of CDA6 protein in *A. flavus*. CDA6 protein was localized in the cell wall of *A. flavus* hypha. The hypha was stained with CFW solution, and the localization of CDA6 protein was observed under laser confocal microscope.

To elucidate the localization of CDA6 protein in *A. flavus* mycelia, we constructed a CDA6-mcherry strain using homologous recombination. Under a laser confocal microscope, the position of red fluorescent in the mycelia of the Δ*cda6* strain indicated the cellular localization of the protein. CFW is a non-specific fluorescent dye that binds cellulose and chitin in the cell wall. The results showed that the red fluorescence of CDA6-mcherry mycelia basically coincided with the blue fluorescence of Calcofluor White (Figure 4), indicating that CDA6 protein was mainly distributed in the cell wall of *A. flavus* mycelia.

### 2.5. cda6 Affects the Sclerotia Formation in A. flavus

**Figure 5:**
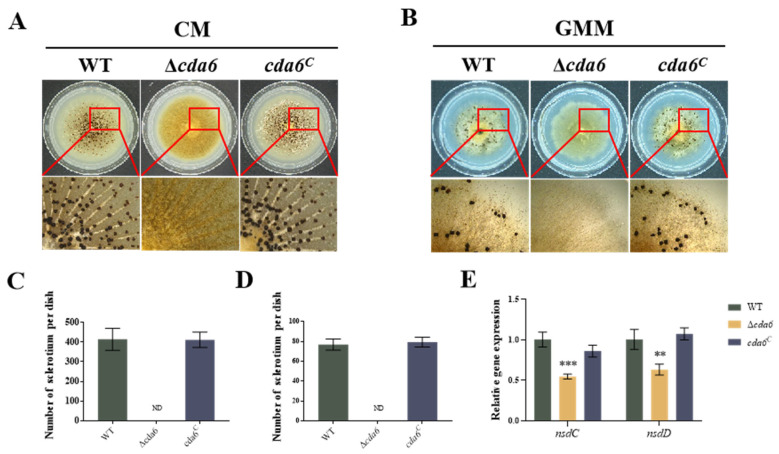
Effect of *cda6* gene on sclerotium formation of *A. flavus*. (**A**) Sclerotium state of WT, Δ*cda6*, and *cda6^C^* strains on CM solid medium, the magnification is 75 times. (**B**) Sclerotium state of WT, Δ*cda6*, and *cda6^C^* strains on GMM solid medium, the magnification is 75 times. (**C**) Sclerotium number of WT, Δ*cda6*, and *cda6^C^* strains on CM solid medium. (**D**) Sclerotium number of WT, Δ*cda6*, and *cda6^C^* strains on GMM solid medium. (**E**) Relative expression levels of *nsdC* and *nsdD* genes in WT, Δ*cda6*, and *cda6^C^* strains. ND indicates no detection, ** indicates that the significance level was *p* < 0.01, and *** indicates that the significance level was *p* < 0.001 (*n* = 3).

To elucidate the role of the *cda6* gene in sclerotia formation in *A. flavus*, the spore suspensions of WT, Δ*cda6*, and *cda6^C^* strains were inoculated with equal concentrations on CM and GMM medium. Statistical analysis revealed that the Δ*cda6* strains did not produce any sclerotia on CM and GMM medium compared to WT (Figure 5A–D), indicating that the *cda6* gene was essential for sclerotia formation in *A. flavus*. To further investigate the mechanism of the *cda6* gene in regulation of sclerotia formation in *A. flavus*, the transcript levels of the *nsdC* and *nsdD* genes related to sclerotia formation were examined by qPCR. The result showed that the relative expression of the *nsdC* and *nsdD* genes in Δ*cda6* strain was significantly lower than that in WT and *cda6^C^* strains (Figure 5E), suggesting that the *cda6* gene regulates sclerotia formation in *A. flavus* by affecting the transcript levels of *nsdC* and *nsdD* genes.

### 2.6. Effect of cda6 Gene on Aflatoxin Biosynthesis

**Figure 6:**
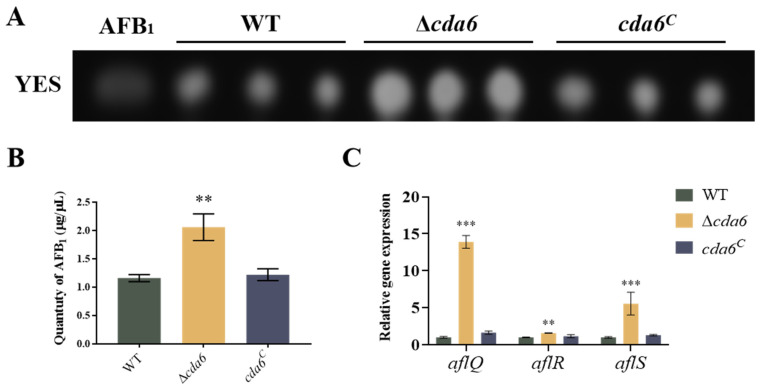
Effect of *cda6* gene on aflatoxin synthesis. (**A**) WT, Δ*cda6*, and *cda6^C^* strains were cultured on YES liquid medium at 29 °C for 6 days, and aflatoxin AFB_1_ was extracted and detected by thin-layer chromatography (TLC). (**B**) Semi-quantitative analysis of aflatoxin in the above strains. (**C**) Relative expression levels of aflatoxin synthesis-related genes *aflQ*, *aflR*, and *aflS* in the above strains. ** indicates that the significance level was *p* < 0.01, and *** indicates that the significance level was *p* < 0.001 (*n* = 3).

To explore whether the *cda6* gene is involved in regulating the biosynthesis of AFB_1_, equal amounts of spore suspensions of WT, Δ*cda6*, and *cda6^C^* strains were inoculated on YES liquid medium, and cultured in the dark at 29 °C for 6 days. Dichloromethane was added to extract the aflatoxin, and thin-layer chromatography (TLC) was used for detection. The results showed that the aflatoxin production of the Δ*cda6* strain significantly increased compared to that of WT and *cda6^C^* strains (Figure 6A,B), indicating that the *cda6* gene negatively regulated aflatoxin synthesis. In order to further explore how the *cda6* gene regulates aflatoxin synthesis, the transcription levels of aflatoxin synthesis related genes *aflQ*, *aflR*, and *aflS* were detected by qPCR. The qPCR data showed that the relative expression levels of *aflQ*, *aflR*, and *aflS* in the Δ*cda6* strain were significantly higher than those in WT and *cda6^C^* strains (Figure 6C), which further proved that the *cda6* gene may negatively regulate aflatoxin biosynthesis by affecting the transcript levels of genes related to aflatoxin synthesis.

### 2.7. cda6 Gene Deletion Affects A. flavus Pathogenicity

**Figure 7:**
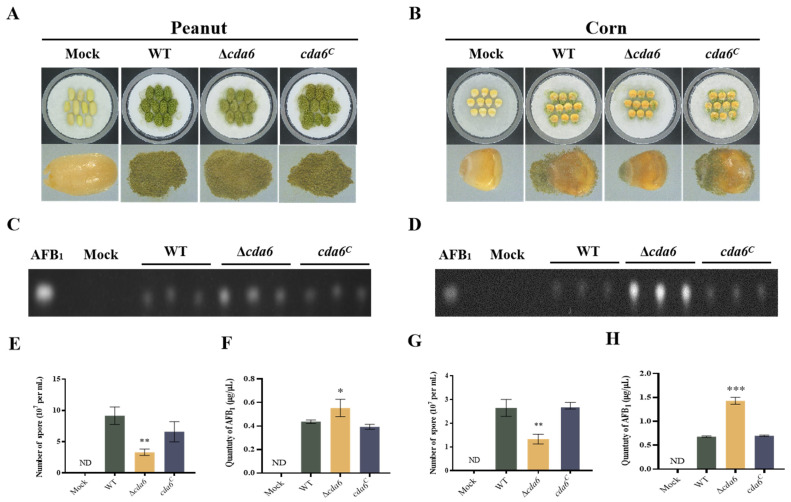
Effect of *cda6* gene on pathogenicity of *A. flavus* to peanut and corn. (**A**) Growth state of peanut seeds infected with WT, Δ*cda6*, and *cda6^C^* strains cultured in the dark at 29 °C for 7 days. (**B**) Growth state of WT, Δ*cda6*, and *cda6^C^* strains on corn kernels cultured in the dark at 29 °C for 7 days. (**C**) AFB_1_ was extracted from peanut seeds and detected by TLC. (**D**) AFB_1_ was extracted from corn and detected by TLC. (**E**) Quantification of conidia from the infected peanut seeds. (**F**) Semi-quantitative analysis of AFB_1_ from peanut seeds. (**G**) Quantification of conidia from the infected corn seeds. (**H**) Semi-quantitative analysis of AFB_1_ from corn. ND indicates not detection, * indicates that the difference was significant *p* < 0.05, ** indicates that the difference was significant *p* < 0.01, and *** indicates that the difference was significant *p* < 0.001 (*n* = 3).

To investigate the role played by the *cda6* gene in infesting peanut and maize seeds, we selected the same batch of peanuts and corn, then infected and cultured them at 29 °C for 7 days to quantitatively analyze conidia and aflatoxin. The data showed that the Δ*cda6*-contaminated peanut and maize produced significantly fewer spores compared to WT and *cda6^C^* strains (Figure 7A,B,E,G). Aflatoxin was extracted and detected by TLC after infestation of peanut and maize, and the results of the semi-quantitative analysis showed that the aflatoxin production from Δ*cda6*-infected seeds was significantly higher than those from WT and *cda6^C^* strains (Figure 7C,D,F,H), indicating that the *cda6* gene affects the pathogenicity of *A. flavus* to crop seeds peanut and maize.

### 2.8. The Role of cda6 Gene in the Stress Response

To investigate the potential involvement of the *cda6* gene in the stress response of *A. flavus*, various stress agents were introduced to the culture medium, including antifungal drugs, SDS, EtOH, and H_2_O_2_. It was observed that the deletion of the *cda6* gene did not result in significant differences in the inhibition rates compared to both the WT and *cda6^C^* strains. This result suggested that the *cda6* gene does not play a discernible role in responding to cell membrane and cell wall stress (Figure S2A–F). Similarly, oxidative stress experimental results revealed no significant variation in the inhibition rate among the WT, Δ*cda6*, and *cda6^C^* strains (Figure S2G,H), implying that the deletion of the *cda6* gene does not impact oxidative stress response in *A. flavus*.

### 2.9. Functional Studies of Structural Domains and Signal Peptide of CDA6 in A. flavus

**Figure 8:**
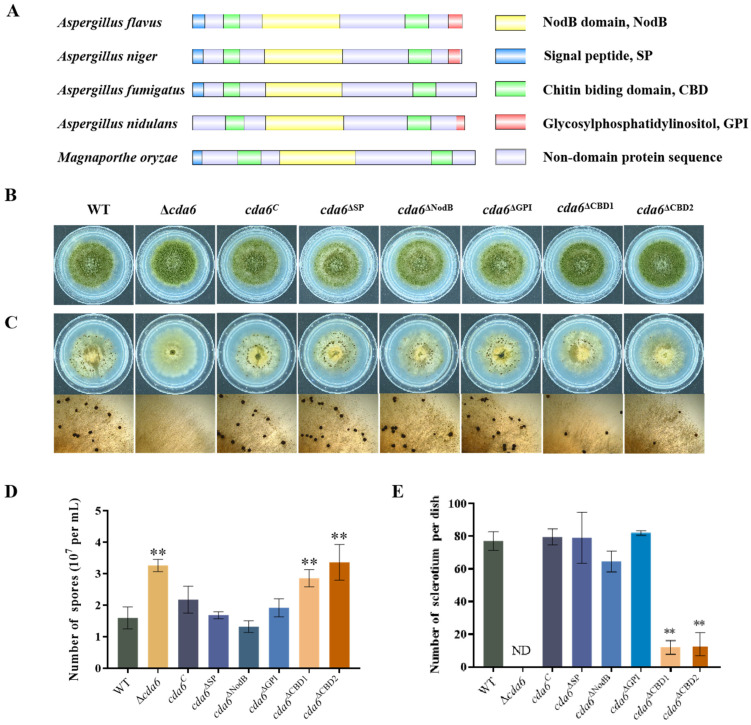
Effects of CDA6 domains and signal peptides on conidial and sclerotium formation in *A. flavus*. (**A**) Domain analysis of CDA homologous proteins in different species. (**B**) WT, Δ*cda6*, *cda6^C^*, *cda6*^ΔSP^, *cda6*^ΔNodB^, *cda6*^ΔGPI^, *cda6*^ΔCBD1^, and *cda6*^ΔCBD2^ strains were inoculated on GMM medium to observe the growth status. (**C**) The sclerotial state of WT, Δ*cda6*, *cda6^C^*, *cda6*^ΔSP^, *cda6*^ΔNodB^, *cda6*^ΔGPI^, *cda6*^ΔCBD1^, and *cda6*^ΔCBD2^ strains after being inoculated on GMM medium and cultured at 37 °C for 10 days. (**D**) The statistics of conidial number from above strains in GMM solid medium (**E**) Statistics of the sclerotium number in GMM solid medium of above strains. ND indicates not detection, and ** indicates that the significance level was *p* < 0.01 (*n* = 3).

The structural analysis of the CDA6 protein in related fungi revealed a conserved structure across different species (Figure 8A). To investigate the role of structural domains and signal peptide of CDA6 in *A. flavus*, domain-deficient strains were generated, including *cda6*^ΔSP^, *cda6*^ΔNodB^, *cda6*^ΔGPI^, cda6^ΔCBD1^, and *cda6*^ΔCBD2^. Growth observation revealed no difference in colony diameter between *cda6* knockout and domain deletion strains compared to WT (Figure 8B), indicating that the structural domains and signal peptides were not involved in regulating the radial growth of *A. flavus*. However, quantitative analysis revealed a significant increase in conidial production in the *cda6*^ΔCBD1^ and *cda6*^ΔCBD2^ strains compared to that in WT strain, while no significant difference was observed in conidial production in the *cda6*^ΔSP^, *cda6*^ΔNodB^, and *cda6*^ΔGPI^ strains (Figure 8D). These findings suggested that the structural domains CBD1 and CBD2 played a major role in the regulation of conidial formation in *A. flavus*. Previous experiments have shown that the deletion of the *cda6* gene causes *A. flavus* to be unable to form sclerotium normally. To investigate the specific effects of various domains and signal peptides of the CDA6 protein on sclerotium formation, WT and all domain deletion mutants were cultured on GMM medium. Compared to the WT, a significant decrease in sclerotial production was observed in the *cda6*^ΔCBD1^ and *cda6*^ΔCBD2^ strains (Figure 8C,E), while no significant changes were noted in other deletion mutants relative to the WT. These results suggested that the CBD1 and CBD2 domains of CDA6 play pivotal roles in regulating sclerotium formation in *A. flavus*.

## 3. Discussion

The fungal cell wall plays a crucial role in the protection, support, and maintenance of cellular morphology, with chitin being a key constituent. The cell wall is a dynamic cell structure, and chitin can be deacetylated to form chitosan, which is catalyzed by chitin deacetylase [25]. In various fungal species, the deacetylation modification mediated by chitin deacetylases is essential for maintaining cell wall morphology, structure, and integrity, particularly in pathogenic fungi [25]. Despite the significance of chitin deacetylation in fungal biology, the specific biological function of chitin deacetylase in *A. flavus* remains largely unexplored.

Previous studies have identified seven chitin deacetylase genes in *A. fumigatus* [19]. Homologs of the *A. fumigatus cda* genes were identified from the *A. flavus* genome and named as *cda1*, *cda2*, *cda3*, *cda4*, *cda5*, and *cda6*, respectively. According to the CAZy (Carbohydrate Active Enzymes database, http://www.cazy.org/, accessed on 15 November 2022), the CDA family is classified within the carbohydrate esterase 4 (CE4) family branch. Through the construction of the evolutionary tree, CDA family members were found to be highly conserved within the genus *Aspergillus*. Additionally, the domains of the CDA family exhibit a high degree of conservation across evolution. Notably, the NodB domain has been identified as functionally related to deacetylation activity [20]. This conservation suggested that the CDA family likely plays a fundamental role in fungal physiology and may have conserved functions across different species within the genus *Aspergillus.*

In this study, we aimed to investigate the function of the chitin deacetylase (CDA) family members in *A. flavus*. We constructed knockout strains for each member of the CDA family, including Δ*cda1*, Δ*cda2*, Δ*cda3*, Δ*cda4*, Δ*cda5*, and Δ*cda6.* We utilized the CFW staining method [26] and a chemical extraction method [27] to quantify the chitin content in the cell wall. Our findings revealed that deletion of individual *cda* genes did not significantly alter the cell wall chitin content or integrity in *A. flavus* (Figure 2), suggesting potential functional redundancy or the involvement of alternative chitin-modifying enzymes. Similar results were reported in *A. fumigatus*, where the deletion of a single *cda* gene or even all seven *cda* genes did not affect fungal cell wall synthesis or virulence. It was revealed that the deacetylation of cell wall chitin in *A. fumigatus* was not catalyzed by members of the CDA family, but probably mediated by other *A. fumigatus* CE4 family deacetylases [19]. The deletion of CDA family members in this study did not affect cell wall integrity in *A. flavus*, and similarly, deletion of the *A. fumigatus* chitin deacetylase *Afcod4* gene did not significantly affect *A. fumigatus* cell wall integrity [18]. In *S. cerevisiae,* two genes (*cda1* and *cda2*) are required for proper ascospore wall formation [14]. In contrast, the results in this study suggested that members of the CDA family may exhibit functional redundancy or may not be directly involved in the deacetylation of chitin; it is plausible that other CE4 family deacetylases could mediate chitin deacetylation in *A. flavus*.

*A. flavus* predominantly disseminates within the ecosystem through the generation of asexual spores, known as conidia. The process of conidiophore production is intricately governed by a network of regulators, including the central regulatory cascade consisting of *brlA*-*abaA*-*wetA* [28]. Our results revealed that the *cda6* gene exerts a negative regulatory influence on conidial formation in *A. flavus*. We observed that the *cda6* gene impacts the transcript levels of key spore formation regulatory genes such as *brlA* and *abaA* during spore development, thereby modulating conidial formation. However, the *cda6* gene did not affect the radial growth of *A. flavus*. However, the *cda6* gene did not affect the radial growth of *A. flavus* (Figure 3). In *A. fumigatus*, the mutant deficient for *cda7* can grow in normal medium, but exhibits reduced conidial production when grown on a medium with GlcNAc as the sole carbon source, indicating a crucial role of *cda7* in GlcNAc metabolism [19]. In the corn fungus *Ustilago maydis*, which contains seven *cda* genes, these genes play critical roles in various aspects of fungal biology, including virulence, adhesion, and plant defense activation [25]. In fission yeast *Schizosaccharomyces pombe*, *cda1* is required for spore formation, and knockdown of this gene results in the formation of a small number of abnormal spores [29]. Additionally, *A. flavus* forms sclerotia, dark brown and hard spherical bodies considered self-protective dormant structures to resist external environments [30]. Regulatory genes *nsdC* and *nsdD* are required for the production of sclerotia in *A. flavus* [31]. In this study, the *cda6* gene is required for the sclerotium formation in *A. flavus* (Figure 5). Meanwhile, the relative expression of *nsdC* and *nsdD* significantly decreased in the Δ*cda6* strain compared to the WT strain. Taken together, these findings suggested that the *cda6* gene plays a multifaceted role in the conidial and sclerotium formation of *A. flavus*.

Previous research has indicated that the distribution of CDA can vary among fungal species, suggesting species-specific localization patterns that may correlate with their respective functions. For instance, in *Absidia coerulea* and *Mucor rouxii*, CDA is distributed in the periplasmic space between the cell wall and the cell membrane [32,33]. Conversely, in *A. nidulans* and *Colletotrichum lindemuthianum*, CDA is secreted into extracellular matrix [34,35]. We hypothesize that the localization of CDA may be related to its specific function within each species. To investigate the localization of CDA6 protein in *A. flavus*, we constructed a cda6-mCherry strain. Our results revealed that CDA6 protein is localized in the mycelial cell wall and has the ability to bind to chitin (Figure 4). Interestingly, despite its presence in the cell wall, CDA6 protein did not exhibit any discernible effects on membrane or cell wall stress response. CDA6 protein in *A. flavus* is highly homologous to CBP1 (chitin-binding protein), a homologous to chitin deacetylase in *Magnaporthe grisea* [24]; we speculated that the function of CDA6 protein in *A. flavus* might be similar to that of CBP1. Considering its localization in the cell wall and its ability to bind to chitin, we hypothesized that CDA6 likely acts as a chitin-binding protein, potentially involved in cell wall integrity.

Aflatoxin, the secondary metabolite of *A. flavus*, includes stable chemical variants such as AFB_1_, AFB_2_, AFG_1_, and AFG_2_, with AFB_1_ as one of the most toxic natural carcinogens [36]. Our findings revealed that *cda6* knockout increased AFB_1_ production. Additionally, we examined the relative expression levels of two aflatoxin production regulatory genes, *aflR* and *aflS*, along with one structural gene, *aflQ*. We observed a significant increase in the expression levels of these genes (Figure 6), suggesting that the *cda6* gene may negatively regulate aflatoxin biosynthesis by affecting the transcript levels of genes related to AFB_1_ synthesis. *A. flavus* is a pathogenic fungus capable of infesting a variety of oilseed crops, such as peanuts and maize, causing significant economic losses [37,38]. Our results showed that the knockout of the *cda6* gene resulted in reduced colonization ability of *A. flavus*. However, the AFB_1_ produced by *A. flavus* in these two oil seed crop species was not reduced (Figure 7). This discrepancy may be attributed to the negative regulatory role of the *cda6* gene in aflatoxin synthesis. Despite the reduced colonization ability of the *cda6* knockout strain, the upregulation of aflatoxin production genes may compensate for the decreased fungal biomass, leading to similar levels of AFB_1_ production in the oil seed crops.

When the external environments changed, *A. flavus* can constantly regulate its own growth and development mechanism to maintain survival. Different antifungal drugs have different mechanisms of action, such as Caspofungin which inhibits the cell wall β-1,3-glycoside synthase, which plays a role in breaking cell wall synthesis [39]. It was reported that the cell wall chitin content largely influenced the sensitivity of the strain to Caspofungin, with higher chitin content decreasing the sensitivity of the strain to Caspofungin [40,41]. Voriconazole inhibits ergosterol synthesis in fungal cell membranes and is used to treat severe fungal infections [42]. Amphotericin B binds to sterols on the fungal cell membrane, thus impairing the permeability of the cell membrane [39]. Our experiments showed that the deletion of *cda6* gene does not affect the sensitivity of *A. flavus* to Caspofungin, Voriconazole, or Amphotericin B, which is different from a previous report (Figure S2A,C). Additionally, we assessed the response of *A. flavus* to cell membrane stress agents such as SDS and EtOH. Our results showed no significant difference in the inhibition rate of the Δ*cda6* strain compared to the WT and *cda6^C^* strains (Figure S2E). Similarly, oxidative stress experimental results showed that there was no significant difference in the inhibition rate between WT, Δ*cda6*, and *cda6^C^* strains (Figure S2G). All above results showed that the *cda6* gene is not involved in the response to cell wall stress, cell membrane stress, or oxidative stress in *A. flavus*.

The signal peptide (SP) and structural domains NodB, GPI, CBD1, and CBD2 are conserved motifs in the CDA6 homologous protein. Previous experiments have demonstrated that the CDA6 protein is located in the cell wall. Initially, we presumed that the localization of this protein might be related to the function of the signal peptide, which guides the transfer and localization of newly synthesized proteins across the membrane. However, our experiments showed that the SP, GPI, and NodB domains are not involved in the regulation of growth and development, AFB_1_ synthesis and pathogenicity of *A. flavus*. While the GPI-anchored structural domain is involved in cell wall integrity and intercellular interactions, and mislocalization of GPI-anchored proteins has a dramatic effect on cell wall composition and fungal virulence in *Candida albicans* [43], our findings suggested that the GPI domain does not play a role in regulating these aspects in *A. flavus*. On the other hand, the chitin-binding domain (CBD) of CDA6 is homologous to the chitin-binding domain of plant chitinase and plant lectins, such as wheat germ lectins (WGA) [44]. In this study, we found that structural domains CBD1 and CBD2 of CDA6 protein are primarily responsible for regulating *A. flavus* development (Figure 8). This suggested that two CBD domains of CDA6 protein play crucial roles in executing its function.

## 4. Conclusions

Our study provides comprehensive insights into the roles of the chitin deacetylase (CDA) family in *A. flavus*. Knockout of individual CDA family members did not impact the chitin content or cell wall integrity of *A. flavus*, suggesting potential functional redundancy within the CDA family or the involvement of alternative CE4 family deacetylases in chitin modification pathways. Moreover, our study identified CDA6 as a key protein localized within the mycelial cell wall. Deletion of the *cda6* gene led to a notable inhibition of conidia formation. Additionally, we observed a critical role for the *cda6* gene in sclerotium formation and its negative regulation of aflatoxin biosynthesis. Moreover, our findings indicated the involvement of the *cda6* gene in the pathogenicity of *A. flavus* towards oil seed crops such as maize and peanut. The structural domains CBD1 and CBD2 emerged as crucial components for the function of CDA6 in *A. flavus* development. In summary, our study showed the diverse functions of the CDA family within *A. flavus*, particularly highlighting the pivotal role of CDA6 protein in various aspects of development, aflatoxin synthesis, and pathogenicity. These findings significantly contributed to our understanding of the biological significance of CDA enzymes in filamentous fungi and offer valuable potential targets for future research and applications in agriculture and biotechnology.

## 5. Materials and Methods

### 5.1. Strains and Culture Conditions

To observe growth and conidial morphology, all strains were cultivated on solid agar media compositions including yeast extract–glucose–trace element (YGT), yeast extract–peptone–sucrose (CM), and glucose minimal medium (GMM) at 37 °C. The GMM medium composition comprises glucose, ammonium tartrate, potassium chloride (KCl), magnesium sulfate heptahydrate (MgSO_4_·7H_2_O), and trace elements [45]. Colony diameters and conidial yields were quantified on the third day post-inoculation. Each experiment was repeated three times.

### 5.2. Sequence Analysis

BLASTP specifically compares a protein query sequence against a protein sequence database to find similar sequences. A BLASTP search was conducted through the National Center for Biotechnology Information (NCBI database, https://www.ncbi.nlm.nih.gov/, accessed on 15 November 2022), and the CDA homologous genes in *A. flavus* were found and named *cda1*, *cda2*, *cda3*, *cda4*, *cda5*, and *cda6*, respectively. Subsequently, BLASTP searches on the NCBI platform were performed to identify homologous protein sequences from diverse species using the conserved domain sequences of the CDA protein family from *A. flavus*. The downloaded protein sequence files were retrieved and imported into MEGA7.0 software for subsequent phylogenetic tree construction, employing the Neighbor-joining method to elucidate the evolutionary relationships among the identified sequences. The Smart (http://smart.embl-heidelberg.de/, accessed on 15 November 2022) and InterPro protein analysis web site (http://www.ebi.ac.uk/interpro/, accessed on 15 November 2022) were employed for comprehensive domain analysis, while DOG 2.0 was utilized for domain mapping. SignaIP 6.0 (https://services.healthtech.dtu.dk/service.php?SignalP-6.0, accessed on 15 November 2022) was conducted to predict the presence of signal peptides within proteins. PredGPI (http://gpcr2.biocomp.unibo.it/predgpi/pred.htm, accessed on 15 November 2022) and TMHMM (https://services.healthtech.dtu.dk/service.php?TMHMM-2.0, accessed on 15 November 2022) were used to perform predictive analysis for the presence or absence of GPI-anchored proteins in proteins.

### 5.3. Construction of Mutant Strains

In this study, the knockout strains of CDA family members were constructed based on the principle of homologous recombination, and experiments were carried out according to a previously described method [46]. Screening marker (*pyrG*) and upstream and downstream homologous fragments were fused and then transferred into the CA14 protoplast of *A. flavus* [47,48]. CA14 was a *pyrG*-deficient strain, which could not grow normally in the medium without exogenous uridine and uracil. To construct the complemented strain, recombinant fragments of the *cda6* gene were introduced into the protoplasts of the *cda6* knockout mutant. Mutant strains with specific domain deletions (such as *cda6*^ΔSP^, *cda6*^ΔNodB^, *cda6*^ΔGPI^, *cda6*^ΔCBD1^, and *cda6*^ΔCBD2^) were also constructed using similar homologous recombination techniques as described above. By fusing mCherry red fluorescent protein as a tag with the target protein, the mCherry fluorescent label strain was obtained, allowing the localization of the protein molecule to be detected. All mutant strains were confirmed by PCR and sequencing.

### 5.4. Calcofluor White (CFW) Staining

An appropriate volume of *A. flavus* spore suspension was inoculated onto sterilized YGT solid medium. Following overnight incubation at 37 °C, mycelium was harvested, washed with phosphate-buffered saline (PBS) solution, and subsequently deprived of culture medium. Fluorescent whitening agent 28 (CFW) was introduced and allowed to incubate under light-protected conditions for 10 min [26]. After incubation, the mycelium was rinsed twice with PBS solution. Subsequently, mycelial samples were visualized under 405 nm excitation light using laser confocal microscopy. Each experiment was repeated three times.

### 5.5. Chitin Quantification

The method of chitin quantification has been reported in previous articles [27]. A standardized volume of *A. flavus* spore suspension was inoculated into YGT liquid medium and allowed to cultivate overnight. Following cultivation, the resultant mycelium was subjected to triple washing with sterile water to eliminate residual culture medium. Subsequently, the mycelium was desiccated, pulverized under liquid nitrogen, and precisely weighed (0.1–0.2 mg). The ground mycelium was then transferred into centrifuge tubes, to which 1 mL of cell wall lysate (comprising 50 mM Tris-HCl, 2% SDS, 0.3 M β-mercaptoethanol, and 1 mM EDTA) was added, followed by incubation at 100 °C for 15 min. To facilitate chitin quantification, an equivalent volume of concentrated hydrochloric acid (HCl) was introduced to acidify the lysate, which was subsequently subjected to drying at 100 °C for 17 h. The application of concentrated HCl serves to hydrolyze chitin and deacetylate the resulting para-acetaminosaccharide to aminoglucan. Chitin was analyzed by measuring the glucosamine released due to acidification using p-dimethylamino benzaldehyde as a chromogen. The filtered supernatant was treated with 4% acetyl acetone reagent, followed by heating at 100 °C for 10 min. Subsequently, 700 μL of 96% ethanol was added to the mixture, which was then supplemented with p-dimethylaminobenzaldehyde reagent and allowed to incubate at room temperature for 1 h. The absorbance of each solution at 520 nm was measured, and analytical data were recorded. Each experiment was repeated three times.

### 5.6. Extraction and Determination of Aflatoxin

A spore suspension with a concentration of 10^7^ spores/mL was inoculated into yeast extract–sucrose agar medium (YES) and incubated at 29 °C for 6 days within a dark environment. Subsequently, the culture solution was subjected to equal-volume extraction with dichloromethane to isolate AFB_1_ via shock extraction. An equivalent volume of dichloromethane was decanted from the lower layer, followed by evaporation to dryness. Dichloromethane was reintroduced to solubilize the toxin, and equal amounts of toxin samples were chromatographed on dry silica gel plates. Thin-layer chromatography was employed for the detection of the AFB_1_, following the procedure detailed in a prior publication [49]. Each experiment was repeated three times.

### 5.7. qPCR Analysis

The experimental procedure for real-time fluorescent quantitative PCR (qPCR) analysis adhered to previously established protocols [50]. RNA extraction was performed using the Total RNA Extraction Kit (TIANMOBIO, Kannapolis, NC, USA), followed by reverse transcription into cDNA. Subsequently, qRT-PCR was conducted using Tip Green qPCR Super Mix (Transgen Biotechnology, Beijing, China) and a PikoReal 96 Real-Time Fluorescence Quantitative PCR System (Thermo Fisher Scientific, Waltham, MA, USA) in accordance with manufacturer’s instructions. Primers targeting the gene of interest were employed for amplification, as delineated in Table S3.

### 5.8. Seed Infections Assay

Seed pathogenicity was assessed following established procedures [50]. Peanut and corn seeds exhibiting uniform morphology were selected for the assay. A blank control (Mock) devoid of infection was served as the control. Seeds underwent sequential washing with sterile water, 0.05% sodium hypochlorite solution, and 75% ethanol. Subsequently, seeds were inoculated with an equivalent concentration of *A. flavus* spores and incubated at 29 °C for 5 days under moist conditions. Following incubation, spores adhering to the seeds were enumerated and processed. Toxin extraction was achieved through thin-layer chromatography. Each experiment was repeated three times.

### 5.9. Stress Response Analysis

Stress response analysis was assessed following established procedures [51]. An appropriate volume of *A. flavus* spore was inoculated in YGT medium plates with different stress agents (0.1 ng/mL Caspofungin, 0.5 ng/mL Voriconazole, 0.1 ng/mL Amphotericin B, 300 µg/mL SDS, 2% EtOH, or 5 mM and 10 mM H_2_O_2_). All the plates were incubated at 37 °C for 5 days. The inhibition rate was calculated as follows: the diameter of the control group minus the diameter of the inhibition group, expressed as a percentage of the diameter of the control group. The stress response experiments were repeated three times.

### 5.10. Statistical Analysis

The data were presented as means (average values) ± SD (standard deviation) of at least three independent biological replicates. GraphPad Prism 8.0 was utilized for data analysis. Statistical significance was evaluated using a one-way analysis of variance (ANOVA). The least significant difference (LSD) test was used as a post hoc test following ANOVA. *p*-values less than 0.05 were considered statistically significant.

## Data Availability

Data are contained within the article.

## Supplementary Materials

The following supporting information can be downloaded at: https://www.mdpi.com/article/10.3390/toxins16050217/s1, Table S1: Information of *cda* homologous gene in *A. flavus*; Table S2: The strains used in this study; Table S3: Primers used for qPCR in this study. Figure S1: Construction of CDA mutants using homologous recombination; Figure S2: Role of *cda6* gene in response of *A. flavus* to stress. Ref. [52] is cited in supplementary materials
