# Supplementary material for: Chitin Deacetylase Homologous Gene cda Contributes to Development and Aflatoxin Synthesis in Aspergillus flavus
Source: Toxins (Basel). 2024 May 9;16(5):217. doi: 10.3390/toxins16050217 (PMC11125919; doi:10.3390/toxins16050217)
Supplement: Supplementary file 1 [file toxins-16-00217-s001.zip › toxins-2827607-supplementary.pdf]

Supplementary Materials: Chitin Deacetylase Homologous Gene *cda* Contributes to Development and Aflatoxin Synthesis in *Aspergillus flavus*

Table S1. Information of *cda* homologous gene in *A. flavus*

Gene name	Gene ID	Accession ID	Amino acid number
cda1	G4B84_000989	XP_041140747.1	609
cda2	G4B84_007455	XP_041147027.1	306
cda3	G4B84_009400	XP_041148937.1	431
cda4	G4B84_005217	XP_041144885.1	247
cda5	G4B84_003875	XP_041143589.1	241
cda6	G4B84_004417	XP_041144085.1	463

Table S2. The strains used in this study

strains	genotype	source
CA14 PTS	Δ*ku70*, Δ*pyrG*	[52]
Wild-type (WT)	Δ*ku70*, Δ*pyrG:: pyrG*	[Prepared in our lab]
Δ*cda1*	Δ*ku70*, Δ*cda1:: pyrG*	this study
Δ*cda2*	Δ*ku70*, Δ*cda2::pyrG*	this study
Δ*cda3*	Δ*ku70*, Δ*cda3::pyrG*	this study
Δ*cda4*	Δ*ku70*, Δ*cda4::pyrG*	this study
Δ*cda5*	Δ*ku70*, Δ*cda5::pyrG*	this study
Δ*cda6*	Δ*ku70*, Δ*cda6::pyrG*	this study
$cda6^{C}$	Δ*ku70*, Δ*cda6::pyrG, cda6::pyrG*	this study
cda6-mCherry	Δku70, Δ*cda6:: pyrG::cda6::mCherry*	this study
$cda6^{\Delta SP}$	Δ*ku70*, Δ*cda6::pyrG*, $cda6^{\Delta SP}$	this study
$cda6^{\Delta NodB}$	Δ*ku70*, Δ*cda6::pyrG*, $cda6^{\Delta NodB}$	this study
$cda6^{\Delta GPI}$	Δ*ku70*, Δ*cda6::pyrG*, $cda6^{\Delta GPI}$	this study
$cda6^{\Delta CBD1}$	Δ*ku70*, Δ*cda6::pyrG*, $cda6^{\Delta CBD1}$	this study
$cda6^{\Delta CBD2}$	Δ*ku70*, Δ*cda6::pyrG*, $cda6^{\Delta CBD2}$	this study

Table S3. Primers used for qPCR in this study

primer	sequence（5'-3'）	remarks
actin-F	ACGGTGTCGTCACAAACTGG	For *β-actin*
actin-R	CGGTTGGACTTAGGGTTGATAG	
cda6-QF	TGCCAAAGGACCCATAGATACAC	For checking whether *cda6*
cda6-QR	GGGCGACCTCATTCCACAGA	is knocked out
brlA-F	GCCTCCAGCGTCAACCTTC	For spore formation related
brlA-R	TCTCTTCAAATGCTCTTGCCTC	genes
abaA-F	TCTTCGGTTGATGGATGATTTC	
abaA-R	CCGTTGGGAGGCTGGGT	
nsdC-F	GCCAGACTTGCCAATCAC	For sclerotium formation
nsdC-R	CATCCACCTTGCCCTTTA	related gene
nsdD-F	GGACTTGCGGGTCGTGCTA	
nsdD-R	AGAACGCTGGGTCTGGTGC	
aflO-F	GTCGCATATGCCCCGGTCGG	For toxin synthesis related
aflO-R	GGCAACCAGTCGGGTTCCGG	genes
aflS-F	AAGCTAAGGCCGAGTCTGG	
aflS-R	CAGGTTGTGTTGCTGTTGATAG	
aflR-F	AAAGCACCCTGTCTTCCCTAAC	
aflR-R	GAAGAGGTGGGTCAGTGTTTGTAG	

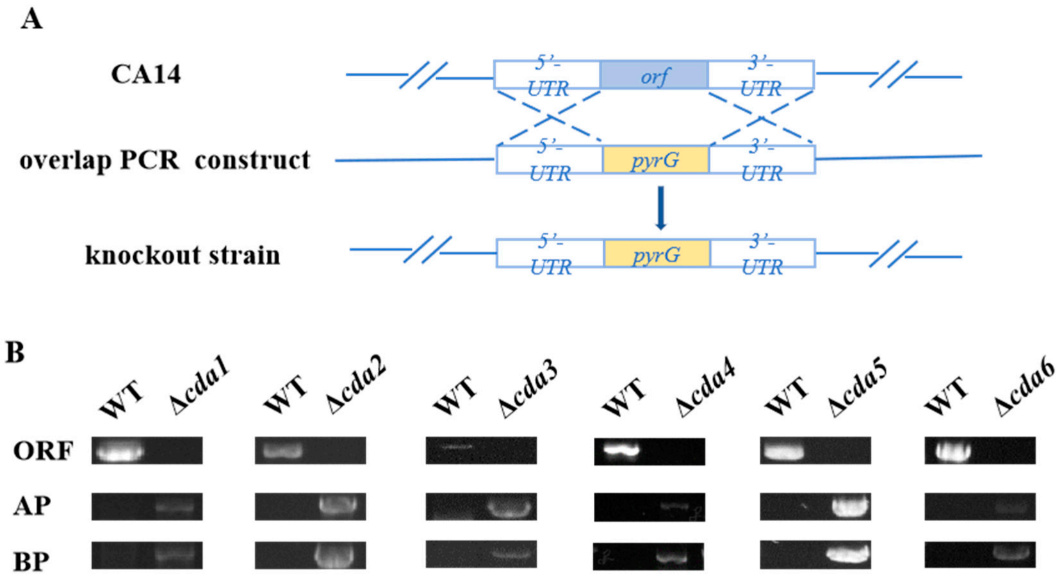

Figure S1. Construction of CDA mutants using homologous recombination. (A) Schematic showing the construction of CDA knockout mutants generated using homologous recombination. (B) Knockout mutations of *cda1*-*cda6* were validated by PCR in *A. flavus*.

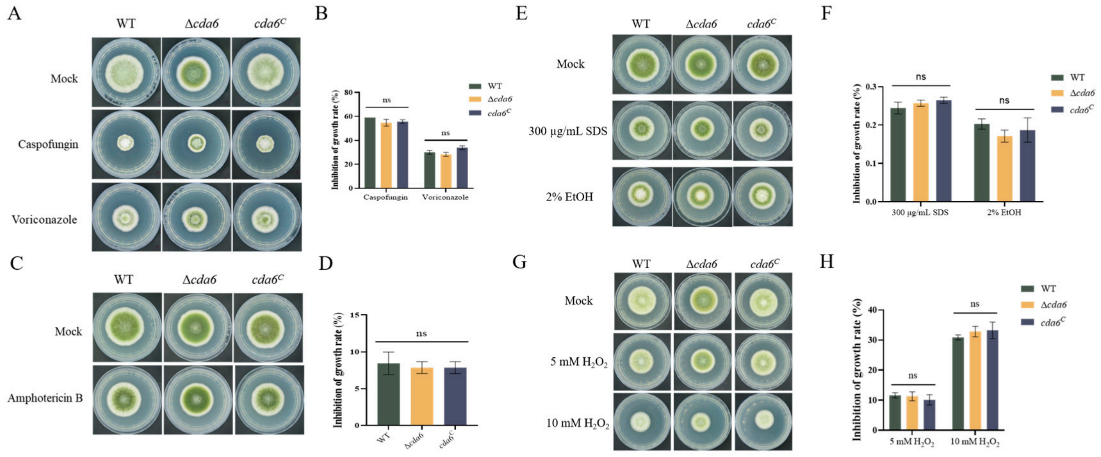

Figure S2 Role of *cda6* gene in response of *A. flavus* to stress. (A) Phenotypes of WT, Δ*cda6*, and *cda6*^{C} strains in media without and with Caspofungin and Voriconazole, respectively. (B) Statistics of the inhibition rate of the above strains in media containing Caspofungin and Voriconazole. (C) Phenotypes of the above strains in media without and with Amphotericin B, respectively. (D) Statistics of inhibition rate of the above strains in media with Amphotericin B. (E) Phenotypes of WT, Δ*cda6*, and *cda6*^{C} strains in YGT medium with 300 μg/mL SDS and 2% EtOH, respectively. (F) Statistics of the inhibition rate of the above strains in media with SDS and EtOH. (G) Phenotypes of WT, Δ*cda6*, and *cda6*^{C} strains in YGT medium with 5 mM and 10 mM H_2O_2, respectively. (H) Statistics of the inhibition rate of the above strains in media with 5 mM and 10 mM H_2O_2. ns indicates no significance ($n = 3$).
